# Neural Coding for Action Execution and Action Observation in the Prefrontal Cortex and Its Role in the Organization of Socially Driven Behavior

**DOI:** 10.3389/fnins.2017.00492

**Published:** 2017-09-07

**Authors:** Stefano Rozzi, Leonardo Fogassi

**Affiliations:** Department of Medicine and Surgery, Unit of Neuroscience, University of Parma Parma, Italy

**Keywords:** behavioral goal, action observation, social interaction, imitation, executive functions, context, monkey

## Abstract

The lateral prefrontal cortex (LPF) plays a fundamental role in planning, organizing, and optimizing behavioral performance. Neuroanatomical and neurophysiological studies have suggested that in this cortical sector, information processing becomes more abstract when moving from caudal to rostral and that such processing involves parietal and premotor areas. We review studies that have shown that the LPF, in addition to its involvement in implementing rules and setting behavioral goals, activates during the execution of forelimb movements even in the absence of a learned relationship between an instruction and its associated motor output. Thus, we propose that the prefrontal cortex is involved in exploiting contextual information for planning and guiding behavioral responses, also in natural situations. Among contextual cues, those provided by others' actions are particularly relevant for social interactions. Functional studies of macaques have demonstrated that the LPF is activated by the observation of biological stimuli, in particular those related to goal-directed actions. We review these studies and discuss the idea that the prefrontal cortex codes high-order representations of observed actions rather than simple visual descriptions of them. Based on evidence that the same sector of the LPF contains both neurons coding own action goals and neurons coding others' goals, we propose that this sector is involved in the selection of own actions appropriate for reacting in a particular social context and for the creation of new action sequences in imitative learning.

## Introduction

**Figure 1:**
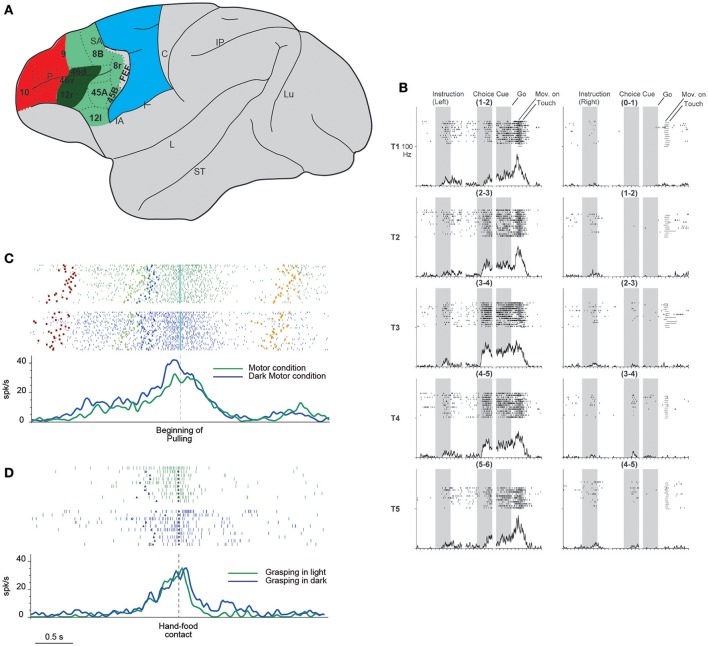
**(A)** Lateral view of the macaque brain showing the parcellation of the prefrontal cortex. LPF is subdivided according to Carmichael and Price (1994), except for its caudo-ventral part (Gerbella et al., 2007). Dashed lines indicate the architectonic borders. The colored shadings delimit, within the frontal cortex, three groups of areas possibly involved in the episodic (red) contextual (green) and sensory (blue) control of executive functions (as defined in humans by Koechlin et al., 2003). This attribution is based on functional and anatomical evidence. In particular, areas in red color have mostly intrinsic prefrontal connections, areas in green are strongly connected with the temporal, parietal, and premotor cortex, the sector in blue corresponds to the premotor cortex. More in details, the sector in dark green is connected with hand-related skeletomotor parietal and premotor areas. C, central sulcus; FEF, frontal eye fields; IA, inferior arcuate sulcus; IP, intraparietal sulcus; L, lateral fissure; Lu, lunate sulcus; P, principal sulcus; SA, superior arcuate sulcus; ST, superior temporal sulcus. **(B)** Example of DLPF neuron showing prominent selectivity for the behavioral goal during choice cue and movement periods. T1–T5 indicate the reached positions; left, and right columns correspond to the trials in which the instructions signal a left or right future target, respectively. Note that each reached position can be to the left or to the right within each pair. When the monkeys is instructed to reach the leftmost target, the activity starts when the behavioral goal is available, and gradually increases toward movement onset, peaking during movement execution. The neuron does not show any activity when the monkey is instructed to reach the rightmost target. Neuronal activity was aligned to the onset of the instruction, choice cue, and GO signal. For each column, the three shaded areas indicate the presentation of the instruction, the appearance and maintenance of the choice cue, respectively (from Yamagata et al., 2012 with permission). **(C)** Example of a VLPF movement-related neuron discharging during grasping in light (green rasters and histogram) and dark (blue rasters and histogram) in a controlled go-nogo task. The neuron discharge begins when the target object is presented and reaches its maximum during grasping execution. Note that no significant difference was present between the two conditions. Rasters and histograms are aligned with the beginning of object pulling. Purple squares: object presentation; blue triangles: release of the starting position; cyan diamonds: beginning of object pulling; orange squares: reward delivery. **(D)** Neural discharge of the same neuron shown in **(C)** during the execution of non-instructed natural reaching-grasping of food. The neuron shows a discharge profile similar to that displayed in the go-nogo task: the response begins slightly before the release of the starting position, peaks during hand-object interaction and ends after grasping accomplishment. Also in this case the neural discharge during grasping does not differ between grasping in light (green) or in darkness (blue). Rasters and histograms are aligned with the hand-food contact (gray circles). Other conventions as in **(C)**. **(C,D)** Modified from Simone et al. (2015).

The lateral prefrontal cortex (LPF) of primates is an anatomically and functionally heterogeneous region that plays a fundamental role in “executive functions”, such as planning, organizing, selecting, and optimizing behaviors based on context and social environment. Neuroimaging studies of humans have suggested that information processing for action planning becomes more abstract when moving from caudal to rostral in the frontal cortex (Fuster, 1997, 2008; Koechlin et al., 2003; Badre and D'Esposito, 2007, 2009; Koechlin and Summerfield, 2007). Koechlin and coworkers, based on fMRI studies in humans, (Koechlin et al., 2003; Koechlin and Summerfield, 2007) suggested that three distinct processes occur: the first, performed by premotor areas, enables the selection of motor responses based on actual sensory stimuli; the second, relying on the caudal LPF, exploits contextual information for the selection of premotor representations; the third, centered on the rostral LPF, participates in the selection of caudal prefrontal representations according to the temporal episode in which the relevant stimuli occur. However, the presence of a hierarchical rostro-caudal organization of the prefrontal cortex is not fully supported by anatomical studies of monkeys focusing on intraprefrontal connections (for a relevant meta-analysis, see Goulas et al., 2014). These data led to a study that reconsidered the LPF's hierarchical organization using an fMRI experiment on humans; the study concluded that the top of the prefrontal processing hierarchy is represented by the middle portion of the LPF (Nee and D'Esposito, 2016). Despite the theoretical differences (partly due to divergent definitions of “hierarchy”), there is general agreement that the core prefrontal region involved in behavior selection and control is located in the central sector of the LPF. This function would require a strict relation between the middle LPF sector and the parieto-premotor circuits subserving sensorimotor transformations. Accordingly, anatomical studies of monkeys have indicated that whereas the rostral LPF (area 10 and the rostral parts of areas 46, 12r, and 9) has mainly intraprefrontal connections, the sector located caudal to it (areas 45A and 45B and the caudal parts of areas 46, 12, and 9) also has strong connections with sensory (e.g., temporal) and sensorimotor (parietal and premotor) areas (Figure 1; Barbas, 1988; Petrides and Pandya, 1999, 2002; Gerbella et al., 2010, 2013; Borra et al., 2011; Saleem et al., 2014).

In addition to the caudo-rostral functional differences, it is known that the dorsal portion of the LPF receives sensory input from the parietal cortex whereas its ventral sector receives information from the temporal cortex. These two types of input are exploited for spatial and object-based working-memory processing, respectively (Levy and Goldman-Rakic, 2000; Passingham et al., 2000).

Although the importance of working memory has been established by many studies, a recent single-neuron study of monkeys (Riley et al., 2016), using simple presentation of visual stimuli, showed that even in the absence of working-memory requirements, it is possible to distinguish a dorso-ventral, and a caudo-rostral subdivision, with posterior areas selective for sensory stimuli and anterior areas involved in more abstract processing.

Finally, research has identified a difference between the left and right human LPF with respect to motor inhibition, such as that required in the decision to act or withhold an action. A meta-analysis showed that the right ventro LPF is specifically active during motor-inhibition tasks but not in reflexive-reorienting tasks (Levy and Wagner, 2011).

Starting from the considerations outlined above, in this review we first discuss the interplay between the LPF and the parieto-premotor circuits for action planning and execution, both in highly demanding cognitive tasks and in basic everyday behaviors. Then, within this context, we discuss the LPF's role in socially driven actions.

## Action coding in the prefrontal cortex

Many studies of the monkey LPF have focused on several aspects of movement planning and the temporal organization of behavior (Fuster, 1997; Saito et al., 2005; Averbeck et al., 2006; Mushiake et al., 2006; Shima et al., 2007; Yamagata et al., 2012; Funahashi and Andreau, 2013; see Miller and Cohen, 2001; Tanji and Hoshi, 2008). The presence of LPF neurons exhibiting movement-related activity has been described mainly in tasks requiring monkeys to make saccades toward specific targets or reach them with the arm (Kubota and Niki, 1971; Niki, 1974a,b; Niki and Watanabe, 1976; Kubota and Funahashi, 1982; Quintana et al., 1988; Funahashi, 1989; Funahashi et al., 1990; Tanila et al., 1992, 1993; Boussaoud and Wise, 1993). In most of these studies, the neural activity *preceding* movement execution was investigated, but few of them have described the activity during *actual execution* (Funahashi et al., 1993; Hoshi et al., 1998).

Yamagata et al. (2012) investigated the role of neurons of the dorsal and ventral LPF (DLPF and VLPF) in movement planning and execution based on arbitrary cues and learned rules. The monkeys were instructed to reach with the arm the rightmost or leftmost target (out of the two presented) based on a previous visual cue. The targets could be presented in six different location pairs. In the first phase of the task, the discharge of VLPF neurons was related mainly to the features of the instruction cues and their behavioral meaning (i.e., the right or left member of the pair). In the subsequent phases, the neurons encoded the spatial location of the target pair specifying the action to be performed. DLPF-neuron discharge was modulated by the behavioral meaning during the period between the giving of an instructing cue and the target's appearance. Interestingly, behavioral meaning modulated DLPF and VLPF neural discharge during movement execution as well (Figure 1B). In the same study, the activity of dorsal premotor (PMD) neurons was compared with that of prefrontal neurons. Most PMD neurons discharge since the beginning of the trials in relation to the behavioral meaning of the instructing cues and continue to discharge until action execution. Thus, this experiment identified a sort of continuum between premotor and prefrontal functions in which, although the premotor cortex has a predominantly motor role and the prefrontal cortex a more abstract role, these roles are partially shared by the two regions.

These results leave open the issue of whether prefrontal neurons are also involved in the control of natural actions. It is well-known that natural actions rely strongly on the sensorimotor transformations provided by parieto-premotor circuits. In particular, the visuomotor integration for reaching is subserved by dorso-dorsal and ventro-dorsal circuits. The former connect superior parietal areas with PMD, the latter inferior parietal areas with the ventral premotor cortex (PMV) (see Rizzolatti and Matelli, 2003). Visuomotor transformations for grasping rely mainly on an inferior parietal-PMV network (see Rizzolatti et al., 2014), although a superior parietal-PMD network also plays a role (see Galletti and Fattori, 2017). Interestingly, both the parietal and premotor nodes of these circuits are also connected with specific prefrontal sectors (see Borra et al., 2017). In phylogenetic terms, these connections may have originally developed in order to control complex behaviors in relation to rich physical and social environments, which require several functions, such as planning distal goals, selection between different alternatives, action sequencing, retrieval from memory, and setting social rules (see Genovesio et al., 2014). In this context, it is likely that the capacity of the prefrontal cortex to deal with abstract functions emerged from the control over the sensorimotor circuits involved in daily behavior.

Seminal evidence of the LPF's involvement in controlling basic sensorimotor functions was provided by Tanila and coworkers (Tanila et al., 1992, 1993), who investigated prefrontal neural properties in the absence of specific tasks and described, in addition to sensory responses, neurons discharging during somatic and eye movements. In particular, somatomotor neurons were concentrated in the middle third of the VLPF, whereas oculomotor neurons were located more caudally.

More recent evidence in this direction was found by Simone et al. (2015), who recorded VLPF neurons in several tasks that, although partly based on learned rules, required monkeys to perform natural reaching-grasping actions. The main task consisted in a go-nogo paradigm, instructed by two differently colored lights. The monkeys had to fixate on the instructing light, and then one of three objects of different sizes and shapes was presented. In the nogo condition, the monkeys had to maintain fixation on the object; in the go condition, they were required, after a delay, to reach for and grasp the object. The reaching-grasping actions were performed in different contextual conditions, e.g., in darkness (in the absence of visual control over hand-object interaction) or on the basis of object memory. Another condition required the monkeys to perform actions in the absence of abstract learned rules; i.e., they could freely reach for and grasp food. The results showed that a specific VLPF sector (including areas 46 and 12) contains neurons that discharge during the execution of goal-directed reaching-grasping actions. Some of these neurons exhibited grip selectivity, and some responded to object observation as well. The movement-related response of these neurons was present in both light and darkness (Figure 1C) and thus was not dependent on visual control over hand-object interaction. They also discharged in a similar way when the object had to be grasped on the basis of memory. Moreover, the authors found that the same neurons that discharged when the monkey was performing the controlled task were active when the monkey grasped food in a natural, as opposed to an instructed, condition (Figure 1D). These data indicate that in addition to implementing abstract rules for complex behavioral responses, VLPF neurons are involved in the control and execution of natural actions. In view of these motor properties and LPF connections with the premotor and parietal cortex, the researchers have compared the properties of the movement-related neurons of these cortical regions. This line of investigation has revealed important similarities in the time course of the discharge, which generally encompasses a motor act (grasping) or even a sequence of motor acts forming the whole action (grasping and pulling). However, there are also important differences: in the VLPF, there are substantially fewer grip-selective neurons than in the parietal and premotor areas, and all the studied VLPF grip-selective neurons lack specificity during object presentation. These differences indicate that VLPF neurons have a marginal role, if any, in visuomotor transformation for grasping.

The above consideration lead to the idea that the neuron discharge during action execution recorded in LPF by Simone et al. (2015) can represent the behavioral goal, namely taking possession of an object. This view is similar to that proposed by previous studies (Hoshi et al., 1998; Saito et al., 2005; Mushiake et al., 2006; Yamagata et al., 2012) based on the learned association between arbitrary stimuli and behavioral outcomes; thus, this understanding of behavioral goals could apply to both natural and learned actions. Within this framework, the VLPF's role in action organization would be keeping active the goal of motor acts represented in the parieto-premotor circuits. These circuits would have a crucial role in the sensorimotor transformations necessary to achieve the goal of the motor acts and, in turn, could provide the VLPF with continuous feedback on goal acquisition during the unfolding of actions. Notably, if the VLPF can access these basic representations of motor goals, it could enable this region to build new actions through learning (see below for a possible mechanism). This conclusion is supported by a recent study (Bruni et al., 2015) in which VLPF neurons were recorded in a task requiring monkeys to perform two different action sequences, i.e., grasping for eating and grasping for placing. The results showed that a higher percentage of movement-related neurons showed a preference for the learned action (grasp-to-place) than for the most natural action (grasp-to-eat).

## Coding of social information in the prefrontal cortex

The prefrontal cortex receives strong visual and auditory inputs from the temporal cortex (Barbas, 1988; Boussaoud et al., 1990; Romanski et al., 1999; Petrides and Pandya, 2002; Saleem and Kondo, 2008; Gerbella et al., 2010, 2013; Borra et al., 2011; Saleem et al., 2014). Among the connected temporal areas, some appear to provide information about biological stimuli. One of the most represented inputs is related to faces, which, as is well-known, are processed in specific temporal sectors (Gross et al., 1972; Perrett et al., 1982; Tsao, 2006; see Tsao and Livingstone, 2008). This input is provided to areas 45 A and 12, where neurons selective for faces have been recorded (Ó Scalaidhe, 1997; Ó Scalaidhe et al., 1999). In these same areas, there are also neurons that discharge while conspecifics' vocalizations are being listened to. Their activity is typically modulated when visual information about the vocalizing face is provided together with the sound (Sugihara et al., 2006; Romanski and Diehl, 2011). The need for this association is demonstrated also by the finding that when the vocalization is congruent with the presented face, the neuron discharge is higher than when the two stimuli are incongruent (Diehl and Romanski, 2014).

Interestingly, it has been shown that neurons in area 45A are related to the production of other monkeys' vocalization. Some of these neurons exhibit mainly a prevocal or perivocal activity and respond also during passive listening to the recorded monkeys calls playbacked (Hage and Nieder, 2015). This suggests that in the monkey prefrontal cortex, at least with respect to vocalization, there is, in addition to multisensory integration, the possibility of audio-vocal integration that could be important for social interactions and, perhaps, lead ultimately to language evolution.

Social interactions rely not only on facial and vocal communication, but also on the understanding of others' actions and the prediction of their outcomes. Thus, if the prefrontal cortex has a role in social interaction, one could predict the presence of neurons sensitive to others' bodily actions. In recent years, it has been shown that this may be the case for brachio-manual actions. Nelissen et al. (2005), in an fMRI monkey experiment, identified a VLPF activation during the observation of grasping actions contrasted with the observation of static controls or scrambled stimuli. The activated areas included areas 46, 45A, and 45B. A similar pattern of activation was recently confirmed by Sliwa and Freiwald (2017).

**Figure 2:**
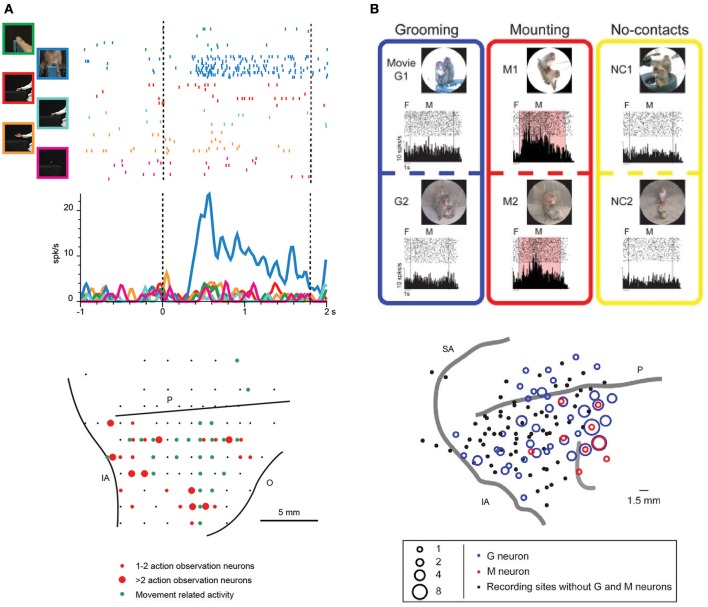
**(A)** *Upper part*: example of VLPF neuron responding the observation of videos depicting biological actions. The neuron discharges exclusively during the observation of a monkey grasping a piece of food from a third person perspective. The vertical dashed lines indicate the beginning and the end of the video presentation. The activity is aligned on the beginning of the video. *Lower part*: distribution of penetrations containing video selective neurons (red circles) in the LPF cortex of a macaque monkey. The green circles represent the penetrations in which movement-related neurons were recorded. The position of the sulci is based on the penetrations depth. O: Orbital reflections. Other abbreviations as in Figure 1. (Modified from Simone et al., 2017). **(B)** *Upper part*: example of a prefrontal neuron responding to the observation of videos depicting mounting behaviors (M1, M2). In this condition the neural discharge is significantly higher than that recorded during the observation of videos showing grooming behaviors (G1, G2) or monkeys not interacting (NC1, NC2). F, fixation period. M, movie period. *Lower part*: location of the sites in which Grooming (blue) and Mounting (red) neurons were recorded in LPF cortex. Abbreviations as in Figure 1. (Modified from Tsunada and Sawaguchi, 2012 with permission).

Recently, monkeys were trained to observe videos showing biological movements and object motion, and VLPF neuronal activity was contemporarily recorded (Simone et al., 2017). Biological movements included goal-directed actions performed by monkeys or humans (e.g., reaching-grasping of food/objects) and non-goal-directed movements performed by humans (e.g., extending an arm or pantomiming the grasping action). The main result was that in the caudal part of areas 12 and 46 and in area 45A, there are neurons responding to the observation of arm movements, the majority of them selectively coding one of the presented stimuli (Figure 2A). Most of these selective neurons discharged more strongly during the observation of reaching-grasping actions. In order to assess whether the neural discharge to action observation was strictly dependent on visual information, a control task was run in which different parts of the videos were obscured. Interestingly, the response of most of the tested neurons was unaffected by the obscuration. Thus, the interpretation of the selective discharge of these neurons was that they mainly code the goal of the action as opposed to its visual description. This type of coding is similar to that proposed for parietal and premotor mirror neurons.

In addition, this study demonstrated that VLPF neurons can code other features of the observed action, such as the agent (human or monkey) or the perspective from which the action was observed (first- or third-person perspective). In fact, the most effective actions were those performed by a monkey, especially from the first-person perspective (40% of the neurons classified as selective for only one stimulus). A slight preference for this perspective was found also in mirror neurons (see below) of the ventral premotor area F5, which were recorded during monkeys' observation of videos depicting grasping actions performed by another monkey from three different perspectives (Caggiano et al., 2011).

Neurons coding others' actions have been widely described in the premotor and parietal cortex. These neurons (mirror neurons) respond to both the observation and execution of motor acts and are thus considered to play a crucial role in action understanding (Gallese et al., 1996; Rizzolatti et al., 1996; Fogassi et al., 2005; Rozzi et al., 2008; Kraskov et al., 2009; Yoshida et al., 2011; Maeda et al., 2015). The VLPF neurons described above (Simone et al., 2017) could be part of a wider action-observation network. In support of this view, a portion of them also discharged when the monkey was performing a grasping action (12% of the visually responsive neurons that were tested also for their motor properties).

The goal-centered coding of VLPF neurons that respond to action observation appears to be related to that proposed for neurons that discharge during reaching-grasping execution. This raises the general question of whether these two categories of neurons could be involved in a common function. Further studies aimed at describing these prefrontal neurons' functional features and their potential differences with the functional features of parietal and premotor mirror neurons will improve our understanding of the VLPF's role in action understanding and response planning.

## Behavioral role of prefrontal neurons related to action observation

Recent studies of the prefrontal cortex have highlighted the importance of social information related to the visual appearance and outcome of others' actions (Tsunada and Sawaguchi, 2012; Falcone et al., 2015; Sliwa and Freiwald, 2017). Concerning single-neuron studies, Tsunada and Sawaguchi (2012) recorded monkey VLPF neurons during the observation of videos showing conspecifics grooming or mounting another monkey and, as a control, the observation of videos in which several monkeys were present but not interacting. They found that 35% of neurons responding during video presentation were selective for grooming or mounting, since they did not respond during the observation of videos depicting non-interacting monkeys (Figure 2B). Notably, the monkeys in this experiment were required simply to observe the scene; thus, it can be concluded that prefrontal neurons respond also when the monkey is not required to exploit the observed stimulus, as was shown by Simone et al. (2017). In another electrophysiological study, Falcone et al. (2015) instructed monkeys to perform a non-matching-to-sample task in which they had to reach a left or right target on a screen, based on the target chosen in the previous trial by themselves or by a human agent. Thus, monkeys had to keep track of the outcome of others' actions to complete the task. In the prefrontal cortex (including areas 46v and 12), they found neurons encoding the agent (monkey or human), the agent's future goal position, and the agent's previous goal position. Among the neurons encoding the human's future goal, some responded to both agents and others only to the human agent's future goal. The authors interpreted the discharge of the latter neurons as a predictive activity concerning the other agent's behavior, and they proposed that the prefrontal cortex is involved in the social-interactive aspects of action coordination and, in particular, in monitoring others' choices.

As a whole, these studies suggest that the prefrontal cortex can use social information to categorize others' behavior and to exploit it in order to guide one's own behavior. The presence in the same region of neurons involved in the observation and execution of goal-directed natural actions is in line with this view. The link between observed and executed actions may lead to the ability to react appropriately to others' actions, e.g., organizing competitive or cooperative actions, or to the ability to reproduce the observed action. The latter capacity is the basis for imitative behavior. Although in adult monkeys there is no clear evidence of true imitation, it is known that they show *facilitation behavior*, which involves an increase in the frequency of an action when witnessing a conspecific performing it (Visalberghi and Addessi, 2000; Ferrari et al., 2005). In facilitation behavior, it is very likely that mirror neurons are involved in the recognition of others' motor acts, but the complex behavioral reaction of the observer is likely guided by another cortical region responsible for the organization of the action sequence. This role may be played by the prefrontal cortex. It should be noted that in humans, imitative learning involves both the parieto-premotor mirror system and area 46 (Buccino et al., 2004).

To sum up, the LPF receives from the parietal and premotor cortex information about the goal of motor acts belonging to one's own motor repertoire and from the temporal cortex visual descriptions of others' actions, and it contains neurons that encode the final behavioral goal (Saito et al., 2005; Falcone et al., 2015). Thus, the visual description of an action can activate the representation of both the behavioral goal and the goals of the motor acts employed to achieve it. This process may be the basis for the selection of own actions suitable for reacting in a particular social context (in both monkeys and humans) and for building new sequences constituted of known motor acts to reach a new final behavioral goal (imitative learning in humans). This proposal requires two further experimental validations: (1) the study of LPF neurons' response during the organization of a behavioral response to others' actions, and (2) the evaluation of the effect of LPF inactivation on behavioral planning based on social context.

## Author contributions

LF and SR critically revised the literature and wrote the paper.

### Conflict of interest statement

The authors declare that the research was conducted in the absence of any commercial or financial relationships that could be construed as a potential conflict of interest.
